# A novel mutation, Ile344Asn, in microsomal triglyceride transfer protein abolishes binding to protein disulfide isomerase

**DOI:** 10.1016/j.jlr.2024.100725

**Published:** 2024-12-12

**Authors:** Swati Valmiki, Cindy Bredefeld, M. Mahmood Hussain

**Affiliations:** 1 Department of Foundations of Medicine, NYU Grossman Long Island School of Medicine, Mineola, NY, USA; 2 Department of Medicine, NYU Grossman Long Island School of Medicine, Garden City, NY, USA

**Keywords:** variants, abetalipoproteinemia, hypobetalipoproteinemia, lipoproteins, apoB

## Abstract

Microsomal triglyceride transfer protein (MTP) plays crucial roles in the assembly and secretion of apolipoprotein B-containing lipoproteins and loss of function MTP variants are associated with abetalipoproteinemia, a disease characterized by the absence of these lipoproteins. MTP is a heterodimeric protein of two subunits, MTP and protein disulfide isomerase (PDI). In this study, we report a proband with abetalipoproteinemia who was monitored annually for 10 years in her third decade and had very low plasma lipids and undetectable apoB-containing lipoproteins. Genetic testing revealed biallelic variants in the *MTTP* gene. She has a well-documented nonsense mutation Gly865∗ that does not interact with the PDI subunit. She also has a novel missense MTP mutation, Ile344Asn. We show that this mutation abrogates lipid transfer activity in MTP and does not support apolipoprotein B secretion. This residue is present in the central α-helical domain of MTP and the substitution of Ile with Asn at this position disrupts interactions between MTP and PDI subunits. Ile344 is away from the known MTP:PDI interacting sites identified in the crystal structure of MTP suggesting that MTP:PDI interactions are more dynamic than previously envisioned. Identification of more missense mutations will enhance our understanding of the structure-function of MTP and the role of critical residues in these interactions between the two subunits. This knowledge may guide us in developing novel treatment modalities to reduce plasma lipids and atherosclerosis.

Dietary and endogenous lipids are transported by apolipoprotein B (apoB)-containing lipoproteins made in the intestine and liver, respectively. ApoB100 synthesized in the human liver is approximately 550 kDa protein and is lipidated co-translationally into a smaller primordial lipoprotein and post-translationally converted into larger lipoprotein by core expansion (1, 2, 3, 4). The assembly and secretion of apoB-containing lipoproteins occurs in the endoplasmic reticulum and is highly dependent on microsomal triglyceride transfer protein (MTP) (2, 5). MTP aids in the lipidation of newly translated apoB protein. In the absence of MTP, newly synthesized apoB100 undergoes intracellular degradation (4, 6, 7, 8, 9). Loss of function biallelic variants of MTP cause abetalipoproteinemia (ABL, OMIM #200100) also known as Familial Hypobetalipoproteinemia due to Secretion Defect 1 (FHBL-SD1) (10, 11, 12, 13). FHBL-SD1 is characterized by the absence of circulating lipoproteins and the presence of pathologies associated with fat-soluble vitamin deficiencies (13, 14, 15). The disease manifests early in infancy with malabsorption and failure to thrive. Without treatment with high-dose fat-soluble vitamins, symptoms advance and include progressive axonopathy, cerebellar dysfunction, and pigmentary retinal degeneration (8). Therefore, there is a need to facilitate early detection of the disease in infancy.

Due to its role in lipoprotein assembly and secretion, MTP inhibitors are used to reduce plasma lipid levels in hypercholesterolemia patients. However, these inhibitors increase lipid retention in liver and intestine (16, 17, 18). Therefore, there is a need to better understand how MTP functions in lipid transfer so that approaches can be made to reduce plasma lipids without causing steatosis. Understanding the structure-function of MTP may provide clues for novel ways of regulating MTP activity.

MTP is a heterodimeric protein consisting of a unique 97 kDa MTP subunit and a ubiquitously expressed 55 kDa protein disulfide isomerase (PDI) subunit (19). MTP is the functional subunit involved in lipid transfer and apoB binding (2, 19). The PDI subunit is essential for the retention of the MTP subunit in the endoplasmic reticulum and also for its lipid transfer activity (20, 21). The MTP subunit has three structural domains; N-terminal β-barrel, central α-helical region consisting of 17 α-helices, and C-terminal lipid transfer domain that contains two β-sheets essential for lipid transfer function (2, 22). The N-terminal domain of MTP may bind to the N-terminal of apoB (23). The central α-helical domain interacts with PDI (22), and the C-terminal domain is involved in lipid binding and transfer (24, 25). Crystal structure studies suggest that subunit interactions between MTP and PDI are mainly hydrophobic and involve the central α-helical domain and C-terminal lipid transfer domain of MTP, and identified Tyr605 as a critical residue for these interactions (22). The structure also highlighted the possible importance of Met600, Asn604, Asp606, and Arg607 in these interactions. Biochemical analysis of a naturally occurring nonsense mutation revealed that C-terminal 30 amino acids are essential for PDI interactions (24). Missense variant analyses highlighted the importance of Arg540 and Asp361 in PDI binding (26, 27, 28, 29).

The phenotype in patients with ABL varies in severity. A major reason could be variable extents of adherence to treatment regimens, dietary, and other socioeconomic factors. Another factor could be genetic. It is possible that different variations in the protein sequence may affect protein activity to different degrees. In this regard, several groups have reported missense mutations in the *MTTP* gene and some of them have been characterized with respect to their effects on MTP function (28, 30, 31, 32, 33, 34, 35, 36, 37). We characterized a few of the reported missense mutations Asp361Try, Arg540His, Asn780Tyr, Ser590Ile, and Gly746Glu and showed that they lacked lipid transfer activities (26). These mutations were in the central α-helical and C-terminal domains of MTP. The Asp361Tyr and Arg540His mutations in the α-helical domain disrupt the MTP:PDI interaction resulting in the loss of MTP functions. We also described a novel mutation in the N-terminal β-barrel of MTP, Asp169Val (38). Molecular studies suggested that Asp169 might be involved in an internal salt bridge and a charge residue at this site is important for protein stability. In addition, we have used site-directed mutagenesis (25) based on crystal structure (22) to identify critical residues in MTP function and found that amino acids with large hydrophobic domains are critical for its lipid transfer function (25).

In the present study, we describe a 41-year-old female proband with two different biallelic variants in the *MTTP* gene. One nonsense variant has been commonly observed in Ashkenazi Jews. She also has a novel mutation in the α-helical domain of MTP which diminishes MTP:PDI interactions, lipid transfer activities, and apoB secretion in human liver-derived cells.

## Materials and Methods

### Materials

Primers used for site-directed mutagenesis were ordered from Integrated DNA Technology, USA. 1,3-Diolein, 2-NBD-X ester (NBD-TG, #6285) was from Setarah Biotech). NBD-phosphatidylethanolamine (NBD-PE, #810145P), L-α Phosphatidylcholine (Egg PC, #131601C), and 1,2-dioleoyl-sn-glycero-3-phosphoethanolamine (# 850725C) were from Avanti Polar Lipids. Rabbit polyclonal anti-MTP antibody was from Abcam (#ab63467) and the rabbit polyclonal anti-β-actin primary antibody was from Cell Signaling (#4967S). Anti-Flag M2 antibody was from Sigma (#F3165), goat polyclonal apoB antibody was from Invitrogen (#PA1-26901), and anti-PDI antibody was from Invitrogen (#MA3-018). Site-directed mutagenesis kit was from NEB (#E0554). Flag peptide was purchased from MedChemExpress (#HY-P0223). The MTP-deficient Huh-7 cells (Mko-3), pcDNA3, and pcDNA3-hMTP-Flag plasmid have been described previously (17, 38, 39).

### Establishment of an ABL and related disorders bioregistry and biorepository and identification of proband

We have established an IRB-approved bio-registry and repository (i23-00665) to collect information about hypobetalipoproteinemia disorders including FHBL-SD1 (10, 11). We contacted members of the Abetalipoproteinemia and Related Disorders Foundation for the identification and referral of patients with hypobetalipoproteinemia disorders. Following participant identification and informed consent, additional clinical and genetic information was requested. One of the voluntary participants was compound heterozygous for a novel missense variant NM_000253.3:c.-1031T > A p.(Ile344Asn) and a common nonsense variant NM_000253.3:c.-2593G > T p.(Gly865∗)in *MTTP* gene. Additional test results including plasma lipid and fat-soluble vitamin levels have been summarized in Table 1. The proband has very low to non-detectable LDL-C, VLDL-C, and non-HDL-C. She had significantly lower levels of total plasma cholesterol and triglyceride levels compared to normal ranges. Additionally, she had low vitamin E and K1 levels, but vitamin A levels were in the normal range. All other parameters were within the expected normal ranges.

**Table 1:** Different blood parameters measured in the proband during her third decade

	Observed Range	Normal Range
Lipids and lipoproteins^a^		
Cholesterol (mg/dl)	36–46	100–199
Triglycerides (mg/dl)	<10	35–150
HDL-C (mg/dl)	27–42	40–80
LDL-C (mg/dl)	ND – <4	50–129
VLDL-C (mg/dl)	ND – 1	16–42
Non-HDL-C (mg/dl)	ND – 4	<130
Vitamins^b^		
Vitamin E (α Tocopherol) (mg/L)	0.4	5.9–19.4
Vitamin E (γ Tocopherol (mg/L)	<0.1	0.7–4.9
Vitamin K1 (ng/ml)	<0.10	0.10–2.20
Vitamin A (μg/dl)	35.2	20.1–62
Vitamin D, 25-hydroxy (ng/ml)	40.2	30–100
Vitamin B12 (pg/ml)	286	232–1245
Folate, serum (ng/ml)	6.0	>3.0
Various blood components^c^		
Total protein (g/dl)	6.7–8.1	6.0–8.0
Albumin (g/dl)	4.0–5.2	3.5–5.2
Globulin (g/dl)	2.5–3.0	2.2–4.2
Alkaline phosphatase (U/L)	33–46	40–130
Aspartate aminotransferase (U/L)	23–54	10–50
Alanine aminotransferase (U/L)	26–77	10–50
Total bilirubin (mg/dl)	0.4–0.9	0.0–1.0
Creatinine (mg/dl)	0.70–0.95	0.5–1.2
Glucose (mg/dl)	93–109	74–106
Hemoglobin A1c (%)	4.7	4.8–5.6
Lipase (U/L)	26–44	12–53
Complete blood cell count^d^		
WBC count (K/μl)	5.9–13.3	3.98–10.04
RDW (%)	13.2–15.1	11.7–14.4
Platelet count (K/μl)	226–318	130–400
Red blood count (M/μl)	4.47–5.31	3.9–5.22
Hemoglobin (g/dl)	10.6–14.9	11.2–15.7
Neutrophil (%)	67.2–79	34–71
Lymphocyte (%)	14.4–23.5	19.3–51.7
Monocyte (%)	4.9–8.4	4.7–12.5
Eosinophil (%)	0.5–5.4	0.7–5.8
Basophil (%)	0.3–1.2	0.1–1.2

ND, not detectable.

a The proband was checked ten times for plasma lipid and lipoprotein levels during 30–39 years of age.

b Vitamin E levels were measured twice and other vitamins were measured once between 39-41 years of age.

c The proband was checked thirteen times for blood components during 35 and 41 years of age. Creatinine and glucose levels were checked six and five times, respectively, during 38–41 years of age. Lipase levels were determined three times. HbA1c was measured once at 39 years.

d The proband was checked eight times for blood cells during 38–41 years of age. Neutrophils were checked three times when she was 38 years old.

The proband was suspected to have ABL when she was eight months old. However, the genetic testing was performed when she was 34 years old. She describes herself as a half Ashkenazi Jew and was adopted at a young age. Her birth father and paternal half-siblings do not have ABL. At the time of this publication, she reported ophthalmologic and neurologic sequelae including nyctalopia and ataxia respectively. Ophthalmic examination and imaging studies demonstrated retinal pigment changes consistent with vitamin A deficiency and angioid streak-like (helicoid-like) peripapillary atrophy; findings previously reported in ABL (40, 41). Electroretinogram testing showed slightly abnormal mixed rod-cone and cone-isolated responses in both eyes. In all, her visual acuity has remained relatively stable. Additional medical history includes hepatic steatosis without reported fibrosis; another common manifestation of ABL (42, 43). Due to a lack of insurance coverage and financial constraints, she does not consistently administer vitamins E and K; however, she takes vitamin A prescriptions that are covered by the insurance.

### Identification of Ile344Asn mutation in the proband

Independent of this study, a clinical specimen from the patient was investigated for mutations in *APOB* and *MTTP* genes using custom next-generation sequencing (NGS) by Fulgent. According to the description provided by the company, genomic DNA was isolated from her blood, barcoded, and enriched for coding regions of *APOB* and *MTTP* genes using hybrid capture technology. Prepared DNA libraries were sequenced using NGS. Following alignment, variants were detected in regions of at least 10x coverage. For both genes, 100% of coding regions and splicing junctions were sequenced with coverage of at least 10x and 20x, respectively, or by Sanger sequencing. The remaining regions did not have 10x coverage and were not evaluated. Variants were interpreted manually using locus-specific databases, literature searches, and other molecular biology principles. All the variants with a quality score of less than 500 were confirmed by Sanger sequencing. Only variants classified as pathogenic, likely pathogenic, or of unknown significance which are thought to be related to the patient’s phenotype or test indication were reported. *APOB* and *MTTP* genes were also evaluated for larger deletion and/or duplication. Identification of a single pathogenic or likely pathogenic variant with autosomal recessive inheritance was ensured by sequencing the 100% coding region of that gene by NGS or Sanger sequencing.

### Generation of plasmids for the expression of Ile344Asn mutation

Primers for site-directed mutagenesis were designed using the NEBase charger tool and the Ile344Asn mutation was created with a Q5 site-directed mutagenesis kit (NEB, #E0554). The forward and reverse primers used for mutagenesis were AAAGAAGAGAACCTTCAAATACTAAAGATGGAAAATAAG and CTTCGCAGTCCTGAG, respectively.

### Lipid vesicle preparation for MTP assay

The donor and acceptor vesicles to study triglycerides (TG) and phospholipid (PL) transfer were prepared as described earlier (44, 45). The donor vesicles for TG consisted of 450 nmol/ml of phosphatidylcholine (PC) and 14 nmol/ml of NBD-TG, while the acceptor vesicles contained 2400 nmol/ml PC, 217 nmol/ml of triolein, and 90 μmol/ml of unlabeled phosphatidylethanolamine (PE). For the PL transfer, donor vesicles were prepared using 400 nmol/ml PC, 90 μmol/ml PE, and 90 μmol/ml NBD-PE. The acceptor vesicles for PL contained PC with a final concentration of 3 mmol/ml. To prepare vesicles, the required amount of lipids was dried under nitrogen gas and dissolved in 15 mM Tris buffer, pH 7.4, containing 40 mM NaCl, 1 mM EDTA, and 0.02% NaN_3_. After proper mixing, the lipid suspension was sonicated (40% amplitude, 60 s pulse on, and 5 s off) using a 550 Sonic Dismembrator (Thermo Fisher Scientific) to obtain a clear solution. The lipid vesicles were then centrifuged at 60,000 rpm or 150,000 *g* using Optima Max-TL Ultracentrifuge with a TLA110 rotor (Beckman Coulter) at 4°C for 1 h. The clear supernatant was collected and 140 mg of NaCl and 20 mg of BSA were added to both donor and acceptor vesicles. The TG donor and acceptor vesicles were mixed and stored at 4°C for further use. The PL donor and acceptor vesicles were stored separately at 4°C and mixed (1:1, v/v) before performing the PL transfer assay.

### Transfection of Mko-3 cells with plasmids expressing MTP

MTP-deficient human hepatoma Huh-7 Mko-3 cells (17) were grown and maintained in Dulbecco’s Modified Eagle Media (DMEM) supplemented with 10% Fetal Bovine Serum (FBS) and L-Glutamine at 37°C and 5% CO_2_ in a humidified incubator. Cells were grown until they reached 80%–90% confluence with media changed at regular intervals. Upon reaching 80% confluence, cells were trypsinized and seeded in 60 mm dishes at a density of 3 × 10^6^ cells per dish. After 24 h, cells were transfected with pcDNA3 control plasmid and pcDNA3-MTP-Flag plasmid expressing either the wild type (WT) or mutant form of MTP using the Endofectin reagent (Genecopoeia #EF013). After 48 h, the media was replaced with fresh DMEM and incubated overnight.

### MTP expression, lipid transfer activity, and apoB100 secretion studies

Transfected Mko-3 cells were harvested in buffer K (1 mM Tris HCL, 1 mM MgCl_2,_ 1 mM EGTA, pH 7.6), lysed by sonication (40% amplitude, 2 s pulse on and 1 s off, for 90 s), and centrifuged at 12,000 rpm or 13,500 *g* (Eppendorf #5424R,) for 10 min at 4°C to remove cellular debris. From the clear supernatant, a small aliquot was used for protein estimation using Pierce BCA method (ThermoScientific #23225). To study cellular MTP expression, 25 μg protein from the clear homogenate was resolved on 8% SDS-PAGE, and the nitrocellulose membranes were probed with an anti-Flag M2 antibody (Sigma #F3165). Rabbit polyclonal anti-β-actin primary antibody (Cell Signaling #4967S) was used to detect the internal reference β-actin. For the TG transfer assay, 25 μg protein of the clear homogenate was incubated with a 5 μl mixture of donor and acceptor vesicles. For blank fluorescence measurements, vesicles were in buffer K. Total fluorescence was obtained by disrupting the vesicles with isopropanol. Percentage of TG and PL transfer was calculated by subtracting the blank, dividing it by total fluorescence, and multiplying it by 100 (44, 45).

ApoB100 secretion was detected in the overnight conditioned media by ELISA (25, 46). Briefly, 96 well plates were coated with 100 μl of the mouse monoclonal anti-hApoB capture antibody 1D1 (MyBiosource, #MBS465020) and overnight incubated at 4°C. The next morning plates were washed three times with PBST buffer (1X PBS with 0.05% Tween 20) to remove any unbound antibody and were blocked with 100 μl of 3% BSA in PBST buffer for 1 h at room temperature. Next, the plates were washed three times and incubated with 100 μl of various concentrations of LDL standards and media samples for 2 h at room temperature. The plates were again washed three times and incubated with 100 μl of goat polyclonal anti-hApoB detection antibody (Invitrogen #PA1-26901) for 1 h, washed three times, and incubated with the alkaline-phosphatase-tagged swine antigoat secondary antibody (SouthernBiotech #6300-04). Finally, the plates were washed and developed by adding 100 μl of p-nitrophenyl phosphate (PNPP) substrate (1 mg/ml) prepared in diethylamine buffer (100 mM glycine, 1 mM MgCl_2_.6H_2_O, and 1 mM ZnCl_2_, pH 10.4). The plates were read at 405 nm, and the concentration of apoB in each sample was calculated with respect to the LDL standards following linear regression kinetics. ApoB protein concentration was normalized with the total intracellular protein concentration.

### PL transfer activity and MTP:PDI interactions

MTP PL transfer activity and MTP:PDI interactions were studied in the purified protein. To purify MTP-Flag, 400 μg protein from the cellular homogenate was incubated overnight at 4°C with anti-Flag M2 affinity gel (Sigma #A2220). Next, the beads were washed three times with buffer K, and finally, the purified MTP protein was eluted with 100 μM Flag peptide (MedChem Express #HY-P0223) in buffer K. For PL transfer assay, protein fraction was incubated with a 10 μl mixture of PL donor and acceptor vesicles and fluorescence was measured every 30 min for 4 h. Blank and total values were measured as described for TG assay. For MTP:PDI interaction studies, the purified proteins were resolved on 8% SDS-PAGE, transferred to nitrocellulose membrane, and probed with anti-hMTP (Abcam #ab63467). The same blot was stripped and re-probed with an anti-PDI antibody (Invitrogen #MA3-018). The lipid transfer activities were normalized with amounts of purified MTP proteins determined from these blots by Image J.

### Statistics

We used GraphPad Prism software (version 9.2.0) for statistical analysis and graphing. To calculate the significance one-way ANOVA nonparametric (multiple comparison) was used, ∗∗∗ and ∗∗∗∗ represent *P* < 0.001 and *P* < 0.0001, respectively. Experiments involving time course as an additional parameter were analyzed using two-way ANOVA. *P* < 0.05 was considered as significant. The bars and error bars represent mean ± SD.

## Results

### Proband

The proband had been regularly tested several times for lipid levels in her third decade (Table 1). She had abnormally low levels of total plasma cholesterol and triglyceride. Her low-density lipoprotein-cholesterol (LDL-C), very low-density lipoprotein (VLDL-C), and non-high-density lipoprotein-cholesterol (non-HDL-C) were in insufficient amounts for proper measurements. However, HDL-C levels were in the normal range. Her vitamin E levels were consistently low when measured at two different times within a year. Similarly, her vitamin K1 levels were low. She had normal levels of vitamin A, 25-hydroxy vitamin D, vitamin B12, and folate. No significant differences were noticeable in various markers of muscle and kidney function. Alanine transaminase (ALT) levels were inconsistently elevated. No significant differences were found in blood cells. In short, these data showed the absence of apoB-containing lipoproteins, and consistently low levels of triglyceride, cholesterol, vitamin E, and vitamin K levels in the proband.

Genetic testing identified two different variants in the *MTTP* gene. The first variant is the well-studied nonsense mutation found in patients with ABL, NM_000252.3:c.2593 G > T (p.Gly865∗) where a premature stop codon is introduced resulting in the synthesis of a truncated protein and loss of function (24). The second variant in the *MTTP* gene NM_000253.3:c.1031 T > A (p.Ile344Asn) has not been previously reported in the Human Gene Mutation Database and in the Broad gnomAD dataset. Analysis of amino acid conservation indicated that the Ile344 is highly conserved (47). This increased the likelihood that an amino acid substitution at this position might not be tolerated. Online predictive algorithms Polyphen (48), Sorting Intolerant From Tolerant (49), PANTHER (50), Rhapsody (51), and alpha-missense (52) predicted the Ile344Asn substitution to be probably damaging (score 1.00), not tolerated, pathogenic, probably damaging (score 0.75) and pathogenic, respectively. These algorithms based on amino acid conservations and neighborhood interactions suggested that this mutation could be deleterious and thus affect protein function.

### Ile344Asn mutant reduces MTP expression and activity

**Fig. 1:**
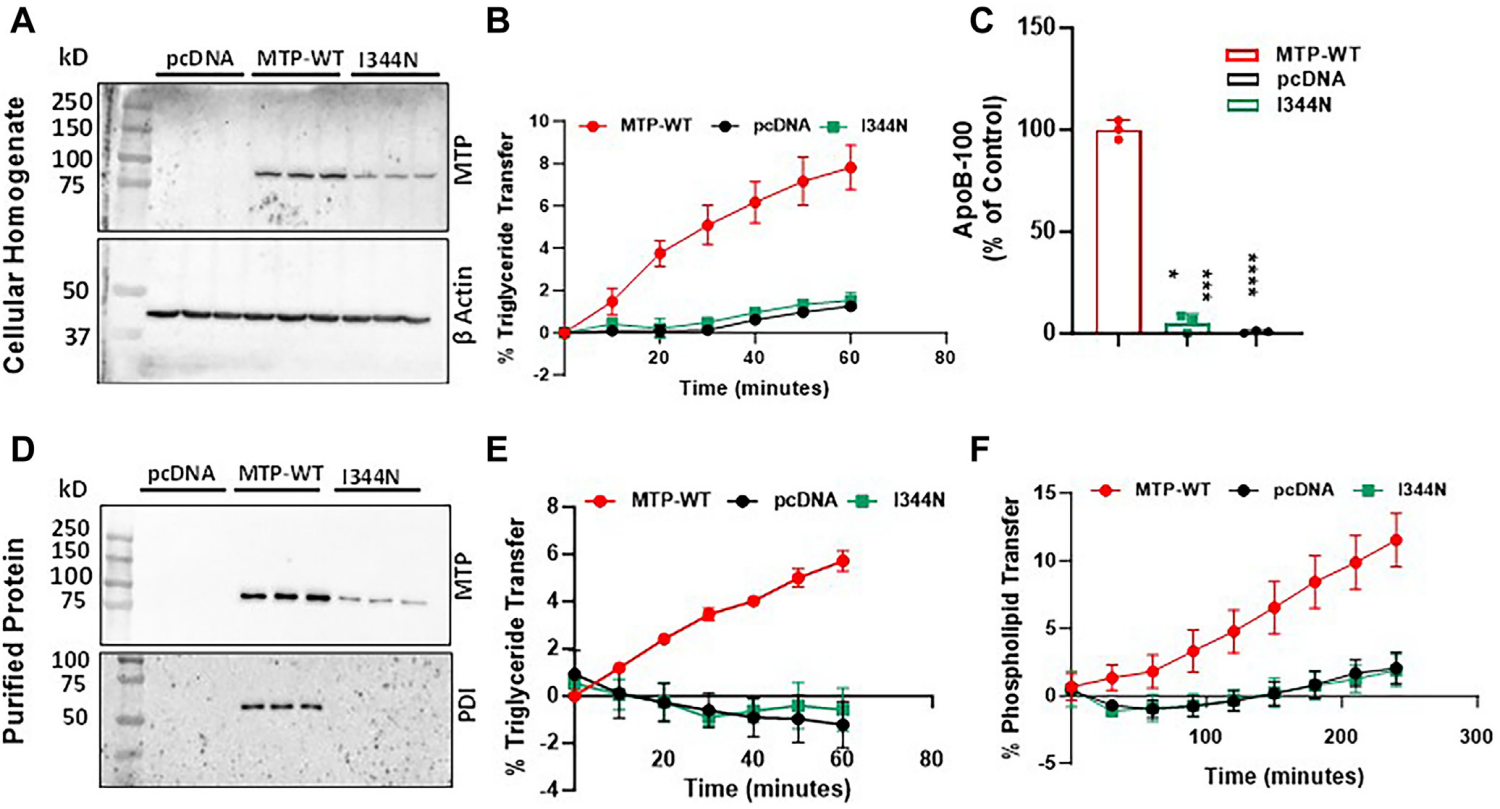
Ile344Asn does not support lipid transfer and apoB100 secretion. A–C: Mko-3 cells were transfected with 3 μg of plasmid expressing either WT or Ile344Asn MTP. After 48 h, media was replaced with fresh DMEM with 10% FBS. After overnight incubation, cell homogenates (25 μg protein) were used to measure MTP and β-actin (control) protein levels (A) by Western blot analysis and to measure triglyceride (TG) transfer activity (B). Overnight-conditioned media was used to measure apoB-100 secreted by the cells by performing ELISA (C). The amounts of apoB were normalized to cell protein levels. D–F: For lipid transfer assay and MTP:PDI interaction studies, 400 μg protein from the cellular homogenate was incubated overnight at 4°C with anti-Flag M2 affinity gel and the purified MTP protein was eluted with 100 μM Flag peptide in buffer K. Purified protein (20 μl) was separated on SDS-PAGE and probed with anti-hMTP antibodies (*top*), stripped and reprobed with anti-PDI antibody (*bottom*) (D). Amounts of MTP protein bands were quantified using ImageJ and values were used to normalize lipid transfer activities. A different aliquot was used to measure TG and PL transfer activities (E–F). The bars and error bars represent mean ± SD. To calculate the significance one-way ANOVA nonparametric (multiple comparison) or two-way ANOVA was used, ∗∗∗ and ∗∗∗∗ represent *P* < 0.001 and *P* < 0.0001, respectively. The data are representative of three independent experiments performed with biological triplicates.

To determine the role of Ile344Asn in MTP function, we generated this mutation in a plasmid expressing human Flag-tagged MTP (25, 26, 38, 39, 53) and performed studies in the human liver-derived Huh-7 cells whose endogenous MTP expression was ablated using CRISPR/Cas9 (17). Consequently, these Mko-3 cells do not secrete apoB100; however, transfection of WT MTP plasmid results in the secretion of apoB100-containing lipoproteins (17). We first transfected the Mko-3 cells with 3 μg of plasmid expressing either the wild-type (WT) MTP or Ile344Asn mutant MTP. pcDNA3 was used as a negative control. Cells transfected with 3 μg of Ile344Asn mutant expressed lower amounts of MTP protein compared with cells transfected with WT-MTP plasmid (Fig. 1A, top). There were no significant differences in β-actin bands indicating equal amounts of proteins were added to different lanes (Fig. 1A, bottom). Cellular homogenates were also used to measure TG transfer activity (Fig. 1B). Cells transfected with plasmids expressing WT MTP showed a robust time-dependent increase in TG transfer activity (Fig. 1B). However, cells transfected with plasmids expressing Ile344Asn variant did not show increased TG transfer activity similar to cells that were transfected with a control pcDNA3 plasmid. Media from these cells was used to quantify secreted apoB. Cells transfected with WT MTP secreted significant amounts of apoB (Fig. 1C). However, cells transfected with mutant Ile344Asn did not secrete apoB similar to those transfected with an empty plasmid (Fig. 1C). These studies indicated that Ile344Asn mutation results in low protein production and cells expressing these mutants do not support TG transfer and apoB secretion.

Next, we purified both WT and mutant MTPs using anti-FLAG columns. Significant amounts of WT MTP protein could be purified using these columns (Fig. 1D, top) and had the PDI subunit (Fig. 1D, bottom). The amounts of Ile344Asn MTP purified were lower than that of WT MTP most likely due to low levels of expression as seen in Fig. 1A. Surprisingly, we did not detect the PDI subunit with the purified Ile344Asn MTP subunit (Fig. 1D, bottom). These studies indicated that Ile344Asn MTP may not interact with the PDI subunit.

We then used purified MTP to assay for its TG and PL transfer activities. Purified WT MTP showed a robust, time-dependent increase in TG and PL transfer (Fig. 1E, F). However, these activities were not measurable in purified Ile344Asn MTP. These studies indicated that the purified mutant protein lacks PDI subunit, lipid transfer activities, and ability to support apoB secretion.

### Increased expression of Ile344Asn mutant MTP did not improve the lipid transfer activity

**Fig. 2:**
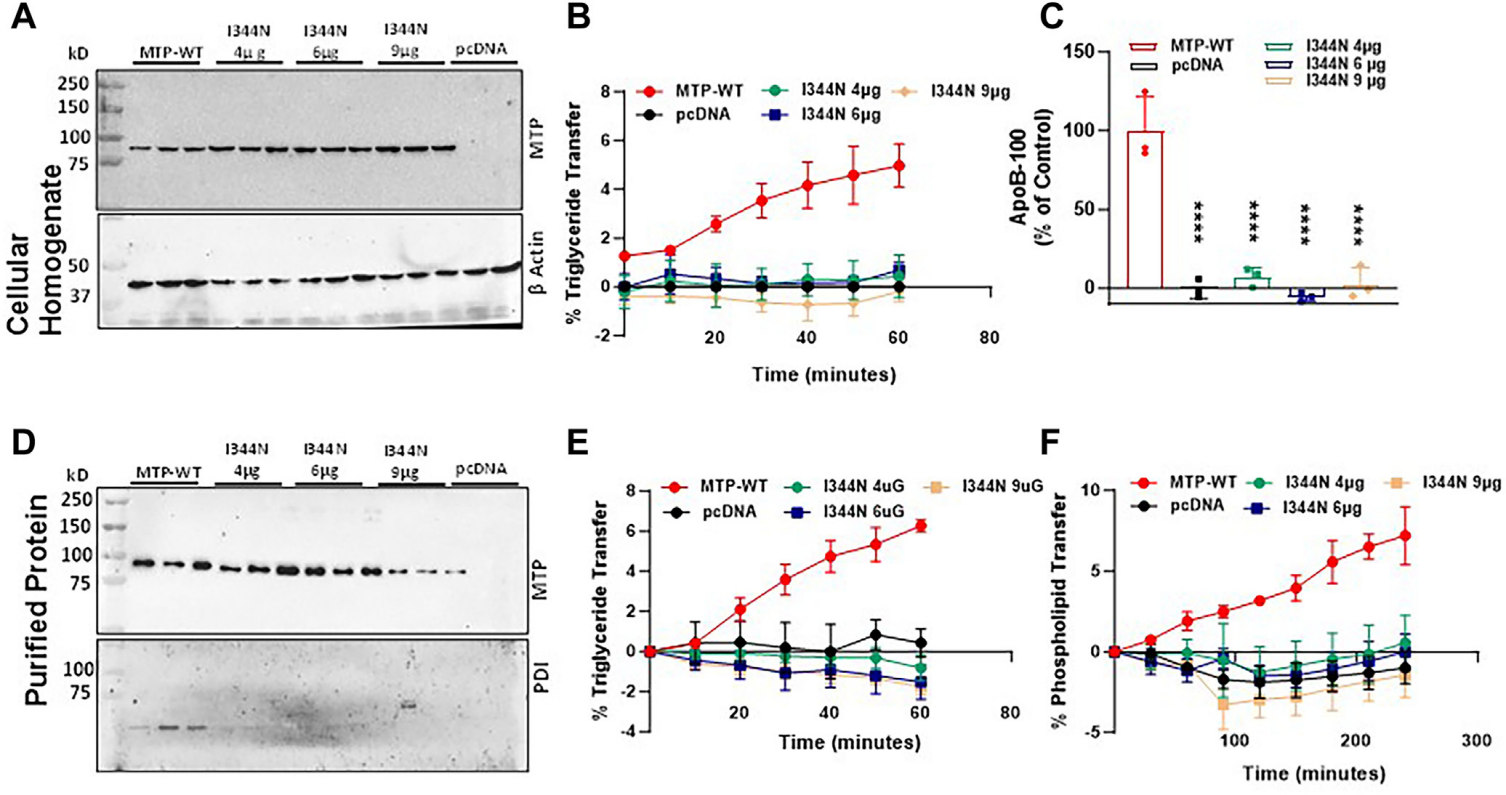
Increased expression of Ile344Asn did not improve the lipid transfer activity and apoB secretion in Mko-3 cell. Mko-3 cells were transfected with either 2 μg of plasmid expressing WT MTP or 4 μg, 6 μg and 9 μg of Ile344Asn MTP. After 48 h, the media was replaced with fresh DMEM with 10% FBS and incubated overnight. The cells were collected, homogenized, and centrifuged at 12,000 rpm or 13,500 *g* for 10 min at 4°C. From the clear supernatant, 25 μg protein was separated on 8% SDS-PAGE and probed with anti-hMTP (A, *top*), stripped, and probed with anti-β-actin antibodies for control (A, *bottom*). For the TG transfer assay, 25 μg protein was used from the clear homogenate (B). Overnight-conditioned media was used to measure apoB-100 secretion by ELISA (C). For lipid transfer assays and MTP:PDI interaction studies, purified proteins were separated on SDS-PAGE and probed for anti-hMTP followed by anti-PDI antibody (D). Different aliquots of purified proteins were used to measure TG and PL transfer activity (E–F). The bars and error bars represent mean ± SD. To calculate the significance, one-way ANOVA nonparametric (multiple comparison) or two-way ANOVA was used. ∗∗∗ and ∗∗∗∗ represent *P* < 0.001 and *P* < 0.0001, respectively. The data are representative of three independent experiments performed with biological triplicates.

The above studies indicated that Ile344Asn lacks lipid transfer and apoB-secreting activities. One caveat of this study was that the cellular amounts of Ile344Asn were significantly lower than the WT protein. Therefore, it was theoretically possible that the negative results observed might be secondary to low expression. Therefore, we attempted to improve the expression of the mutant protein. To achieve equal MTP expression we transfected Mko-3 cells with 2 μg of plasmid expressing the WT MTP and increasing amounts of plasmid expressing Ile344Asn MTP. Transfection of cells with higher amounts of plasmid led to increased expression of the Ile344Asn mutant MTP (Fig. 2A). However, the increased MTP expression did not improve the TG transfer activity. Cell lysates expressing different amounts of mutant MTP showed no TG transfer activity similar to that seen in control pcDNA3 transfected cells (Fig. 2B). Cells transfected with WT MTP supported apoB secretion into the media. In contrast, no apoB could be detected in media of cells transfected with different amounts of mutant MTP (Fig. 2C). These studies indicate that mutant Ile344Asn is deficient in TG transfer activity and does not support apoB100 secretion in Mko-3 cells and that this deficiency was not due to low protein expression.

### Ile344Asn does not interact with PDI

Next, we purified WT and mutant MTP from these cells (Fig. 2D). The amounts of purified protein obtained from cells transfected with 4 and 6 μg of plasmid expressing the Ile344Asn mutants were similar to those of purified WT MTP protein. The purified WT MTP contained PDI subunit; however, the PDI subunit was not seen in the purified Ile344Asn mutant MTP (Fig. 2D). We used the purified proteins to assess TG and PL transfer activities. Purified WT MTP showed robust TG and PL transfer activities (Fig. 2E, F). However, purified mutant MTP did not show any TG and PL transfer. These findings suggest that the substitution of Ile with Asn at the 344 position abolishes the MTP binding with the PDI subunit and MTP transfer activities.

### Subtle structural changes in MTP structure due to Ile344Asn mutation

**Fig. 3:**
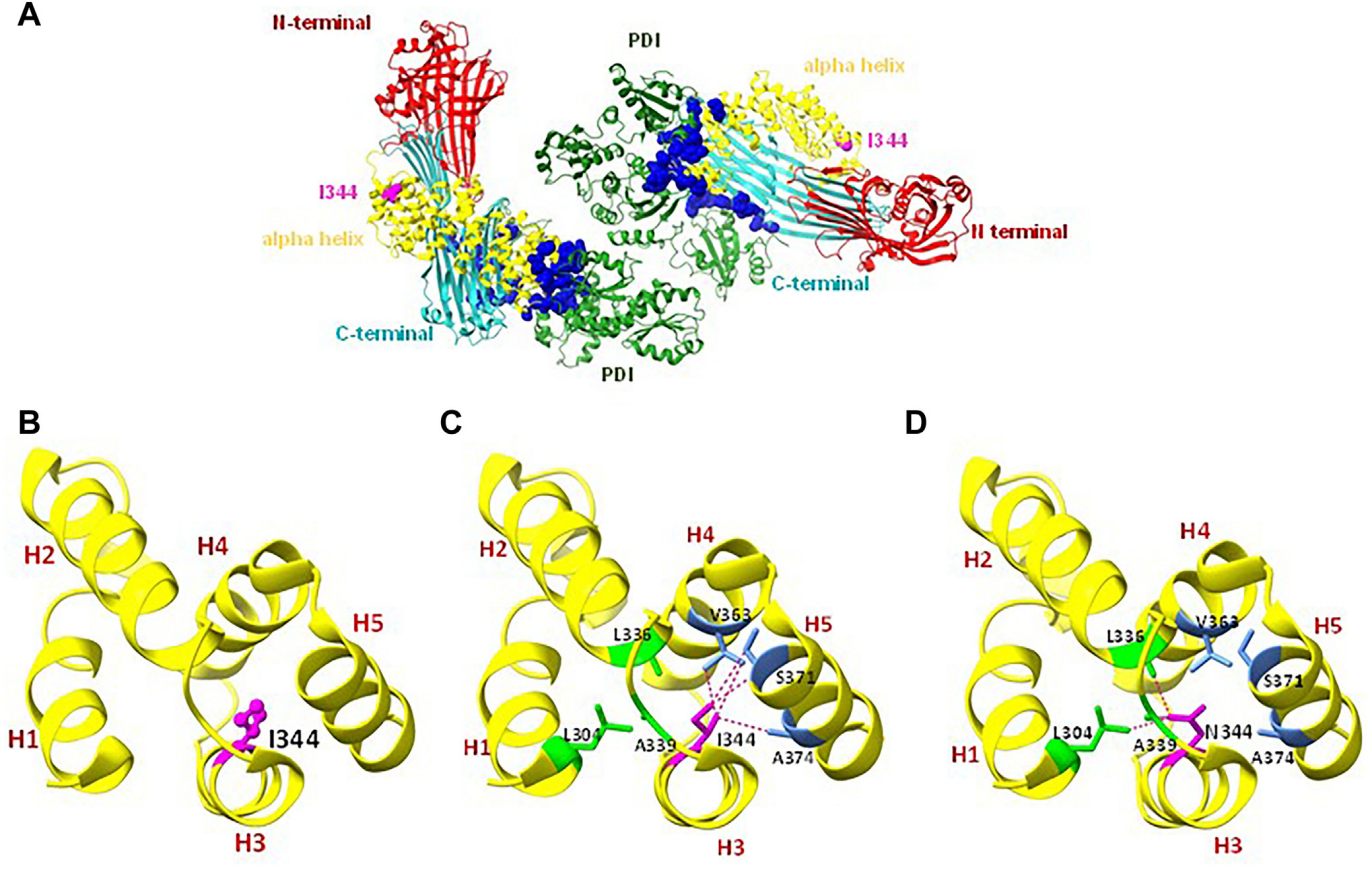
Substitution of Ile with Asn at 344 may disrupt interactions between helices within the central α helical domain of MTP. A: Ile344 is identified in the MTP:PDI crystal structure. Ile344 is away from the predicted MTP:PDI interacting interface (colored in *blue*). B: Ile344 is present in the third stand of the central α helical domain. C: Ile344 interacts with Val363 on Helix 4 and Ser371 and Ala374 of helix 5. D: Ile was replaced with Asn using the rotamer command in Chimera X and its interactions with nearby amino acid were studied. In contrast to Ile344, Asn344 appears to interact with Leu304 in helix 1, Leu336 in helix 2, and Ala339 residue present in the connecting coil between helices 2 and 3.

Next, we used Chimera X software (54, 55) to gain insights into possible alterations the Ile344Asn mutation might have caused in MTP structure. While the hydrogen bonds between the carbonyl oxygen of one amino acid and the amine group of every fourth amino acid are the primary forces that maintain individual α-helix structure, the side chain interactions between helices also contribute to the proper folding and stability (56, 57, 58). Therefore, we identified amino acid residues present within 4 Å of the Ile344 residue and looked for possible interactions with nearby amino acid side chains. Ile344 is in the third helix of the central α-helical domain that consists of 17 α-helices. This region is located at a distance from the predicted PDI interacting site which is present in the helix 17 of the central helical domain and C-terminal lipid binding region (Fig. 3A, B). First, every connection between Ile344 and nearby amino acids was displayed at a distance of 4 Å. Ile344 side chains were predicted to interact with the side chains of Val363 on helix 4 and Ser371 and Ala374 on helix 5 (Fig. 3C, supplemental Video S1). We then replaced Ile with Asn using the rotamer command and selected the rotamer with the highest probability according to Dunback’s library (59). When Ile was replaced with Asn, it was predicted to interact with Leu304 on helix 1, Leu336 on helix 2, and with Ala339 which is present in the connecting loop between helix 2 and helix 3 (Fig. 3D, supplemental Video S2). From these structural analyses, we propose that Ile344Asn might have caused subtle changes in the amino acid interactions in this region which altered the interactions between neighboring helices. This might be responsible for the loss of MTP:PDI interactions in this mutant.

### Analyses of Asp361Tyr and Arg540His missense mutations suggest ionic and hydrophobic interactions amongst different helices within the α-helical domain of MTP might be important for PDI binding

**Fig. 4:**
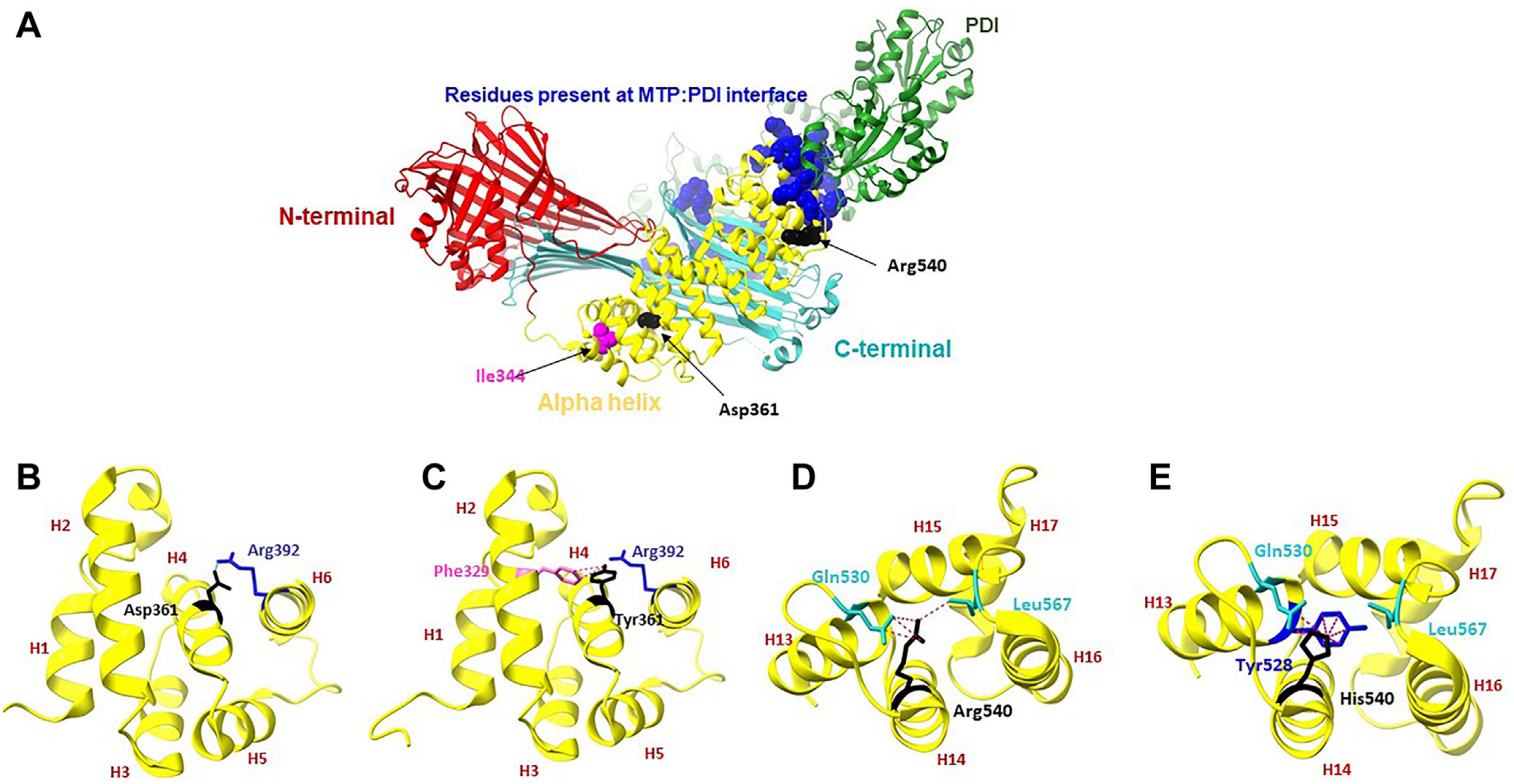
Possible explanations for the lack of PDI binding in Asp361Tyr and Arg540His missense mutations. A: Asp361 and Arg540 are identified in the MTP:PDI crystal structure. Asp361 is away from the predicted MTP:PDI interacting interface while Arg540 is present in helix 14 close to the MTP:PDI interacting site (colored in *blue*). B: Asp361 is present in the fourth strand of the central α helical domain and interacts with Arg392 on Helix 6. C: Tyr361 which instead appears to interact with Phe329 in helix 2. D: Arg540 in helix 14 is predicted to interact with the side chains of Gln530 and Leu567. E: When replaced with His, it was predicted to interact with Tyr528 in helix 13.

We had previously characterized Asp361Tyr, Asp384Ala, Arg540His, and Ser590Ile ABL missense mutations in the α-helical domain of MTP (26, 39). Asp384Ala and Ser590Ile mutations did not affect MTP:PDI interactions, while the Asp361Tyr and Arg540His mutations disrupted these interactions. Similar to Ile344, both Asp361 and Arg540 are not at the hydrophobic MTP:PDI interacting site observed in the crystal structure. Therefore, we performed Chimera X analysis to identify the structural basis for the loss of subunit interactions in these mutants. The Asp361 residue is close to the Ile344 in helix 4 of the central α-helical domain, while the Arg540 is in helix 14 close to the PDI:MTP interaction site (Fig. 4A). Our analysis revealed that the negatively charged Asp361 is in close proximity to positively charged Arg392 in helix 6. Tyr361 was instead predicted to interact with an aromatic amino acid, Phe329 in helix 2 (Fig. 4B, C, supplemental videos S3 and S4). Thus, Asp361Tyr is likely to disrupt the salt bridge between Asp361 and Arg392 and destabilize the tertiary structure of MTP.

We next looked for amino acids within 4 Å of Arg540 to identify possible ionic interactions, but we did not find any charged residue. Instead, Arg540 was in close proximity to the side chains of Gln530 and Leu567. Gln530 is in the coil region that connects helices 13 and 14, whereas Leu567 is in the coil region that connects helices 16 and 17 (Fig. 4D, supplemental Video S5). When Arg540 was replaced with His, it was predicted to interact with Tyr528 in helix 13 (Fig. 4E, supplemental Video S6). Thus, analyses of these missense mutations suggest that the tertiary structure of the α-helical domain of MTP is stabilized by both ionic and hydrophobic interactions to maintain interactions with the PDI subunit.

## Discussion

Over the ten-year period of observation, the proband consistently had significantly low levels of cholesterol and triglyceride. The amount of apoB-containing lipoproteins was too low to measure at all-time points. Vitamin E and vitamin K levels were significantly lower than normal values. These features point to the presence of either FHBL-SD1 (ABL) or FHBL-SD2 homozygous hypobetalipoproteinemia. Genetic testing identified two different variants in the *MTTP* gene but no variants in the *APOB* gene. Therefore, this proband is a classic example of FHBL-SD1.

The two different variants in the *MTTP* gene found in the proband include one allele with a nonsense mutation that results in the truncation of the protein at Gly865. This is a very common mutation found in the Ashkenazi Jewish population with a carrier frequency of 1 in 131 (60). This mutant protein does not interact with PDI and is unable to transfer lipids. Homozygosity of this mutation has been shown to be associated with very low levels of plasma TG and cholesterol (24). The second variant, Ile344Asn, has not been previously reported to our knowledge. The genetic test results described this variant to be of unknown significance. Therefore, we undertook studies to determine whether this mutation is pathogenic or not. Our studies show that Ile344Asn missense mutation is pathogenic and results in the loss of PDI binding, lipid transfer activities, and ability to support apoB secretion. We propose that the loss of lipid transfer activities in the MTP subunit is most likely secondary to its inability to interact with the PDI subunit. These studies are consistent with the observations that disruptions of MTP and PDI interactions using chemical and biophysical methods result in loss of MTP lipid transfer activity (20, 21). Since the mutant MTP could not transfer lipids, it is likely that it was unable to lipidate nascent apoB and, therefore, apoB was not secreted imparting a phenotype consistent with ABL in this patient.

Several mutations in MTP have been shown to result in loss of PDI binding. Ricci *et al.* characterized a nonsense mutation, G865∗ (the same missense mutation also found in this patient), that results in the loss of C-terminal 30 amino acid in the MTP subunit. This truncated protein did not bind PDI indicating that PDI interacting site might be in the C-terminus of the molecule (24). Nevertheless, the characterization of different missense mutations in other patients with ABL highlighted the importance of specific residues in the central α-helical domain in PDI binding. For example, Arg540His and Asp361Tyr mutations decrease PDI binding (26, 27). The Arg540 residue is located in the helix 14 close to the predicted MTP:PDI interacting site and the positive charge at this position could be crucial for maintaining the MTP:PDI interaction. The Arg540His disrupts the MTP interaction with PDI while the Arg540Lys does not. Rehberg *et al.* suggested that this is due to the difference in the pKa for ionizable guanidino nitrogen of arginine and imidazole nitrogen of histidine. Since the side chains of Arg and Lys possess similar pKa, the Arg540Lys does not disrupt the MTP:PDI binding and MTP lipid transfer activity (28). Our data suggest that Arg540 may interact with Gln530 and Leu567 via hydrophobic interactions. Thus, these interactions may be important to stabilize the tertiary structure of the protein.

Ile344 and Asp361 are present in helices 3 and 4, respectively, which are located at a distance from the MTP:PDI interacting site. Therefore, it was surprising to observe that these mutations abolished PDI binding. Our studies suggest that Ile344 interacts with residues in other helices via hydrophobic interactions. In contrast, Asp361 forms a salt bridge with Arg392. Thus, residues in helices 3 and 4 most likely interact with residues in other helices via both ionic and hydrophobic interactions. All of these interactions are important for MTP activity and PDI binding. Therefore, it is likely that the helices around these residues are not directly involved in PDI binding and that these mutations cause structural instabilities altering the PDI interacting domains.

Not all ABL mutations disrupt PDI binding. Several other ABL mutations in the α-helical domain (Try528His, Leu435His, and Ser590Ile) do not affect PDI binding. Therefore, known mutations can be categorized into two groups; mutations that disrupt PDI binding and those that do not affect PDI binding. Mutations that do not affect PDI binding can be further subdivided into residues that are directly involved in lipid binding and transfer (25) and others that are not involved in lipid transfer (25) but play a critical role in stabilizing the tertiary structure of MTP (38) and therefore are indirectly critical in the lipid transfer function.

In summary, the analysis of mutations in the proband and other patients highlighted the importance of interactions between MTP and PDI subunits. The nonsense mutation Gly865∗ has been described to abolish MTP:PDI binding. Our studies show that the other variant found in this patient, lle344Asn, cannot also interact with PDI. Therefore, this patient appears to have two different pathogenic mutations that destabilize MTP:PDI interactions.

## Data availability

All the data are in the manuscript.

## Supplemental data

This article contains supplemental data.

## Conflicts of Interest

The authors declare that they have no conflicts of interest with the contents of this article.

## Supplemental data

Movie 1 Interactions of Ile344 with neighboring amino acids.

Movie 2 Possible interactions of Asn344 with neighboring amino acids.

Movie 3 Interaction of Asp361 with Arg392.

Movie 4 Interactions of Tyr361 with neighboring amino acids.

Movie 5 Interactions of Arg540 with neighboring amino acids.

Movie 6 Interactions of His540 with neighboring amino acids.
